# *CFTR* variants and renal abnormalities in males with congenital unilateral absence of the vas deferens (CUAVD): a systematic review and meta-analysis of observational studies

**DOI:** 10.1038/s41436-018-0262-7

**Published:** 2018-09-14

**Authors:** Hongcai Cai, Xingrong Qing, Jean Damascene Niringiyumukiza, Xuxin Zhan, Dunsheng Mo, Yuanzhong Zhou, Xuejun Shang

**Affiliations:** 1 Family Planning Research Institute/Center of Reproductive Medicine, Tongji Medical College, Huazhong University of Science and Technology, Wuhan, Hubei China; 2 Department of Gynecology, Jiangmen Central Hospital, Affiliated Jiangmen Hospital of Sun Yat-sen University, Jiangmen, Guangdong China; 3 Department of Reproductive Medicine, Xi’an No. 4 Hospital, Xi’an, Shaanxi China; 4 Department of Urology, Liuzhou Worker’s Hospital, Fourth Affiliated Hospital of Guangxi Medical University, Liuzhou, Guangxi China; 5 School of Public Health, Zunyi Medical University, Guizhou Zunyi, China; 6 Department of Andrology, Jinling Hospital Affiliated to Southern Medical University, Nanjing, China; 7 Department of Andrology, Jinling Hospital, Nanjing University School of Medicine, Nanjing, China

**Keywords:** congenital unilateral absence of the vas deferens, *CFTR*, F508del, 5T, renal abnormality

## Abstract

**Purpose:**

*CFTR* variant is the main genetic contributor to congenital (unilateral/bilateral) absence of the vas deferens (CAVD/CUAVD/CBAVD). We performed a systematic review to elucidate the genetic link between *CFTR* variants, CUAVD, and the associated risk of renal abnormality (RA).

**Methods:**

We searched relevant databases for eligible articles reporting *CFTR* variants in CUAVD. The frequency of *CFTR* variants and RA, and the odds ratios (ORs) for common alleles and RA risk, were pooled under random-/fixed-effect models. Subgroup analyses and heterogeneity tests were performed.

**Results:**

Twenty-three studies were included. Among CUAVD patients, 46% had at least one *CFTR* variant, with 27% having one and 5% having two. The allele frequency in CUAVD was 4% for F508del and 9% for 5T. The summary OR for 5T risk in CUAVD was 5.79 compared with normal controls and 2.82 compared with non-CAVD infertile males. The overall incidence of RA was 22% in CUAVD. The pooled OR for RA risk among CUAVD patients was 4.85 compared with CBAVD patients.

**Conclusion:**

*CFTR* variants are common in CUAVD, and the 5T allele may be associated with increased CUAVD risk. CUAVD patients bear a higher RA risk than CBAVD patients, but this is not associated with *CFTR* variants.

## Introduction

Congenital absence of the vas deferens (CAVD) is a common urological disease probably caused by defects of the Wolffian ducts and contributing to obstructive azoospermia (OA).^1^ CAVD is classified into three subtypes: congenital bilateral absence of the vas deferens (CBAVD), congenital unilateral absence of the vas deferens (CUAVD), and congenital bilateral partial aplasia of the vas deferens (CPAVD).^2^ CBAVD is the most common subtype, accounting for 1–2% of infertile but otherwise healthy males and up to 25% of OA cases.^3^ CUAVD, with a prevalence of 0.5–1.0% in males, is usually discovered during evaluations for infertility or surgical procedures of the male genitalia.^4^ However, the incidence of CUAVD could be underestimated due to the possibility of pregnancy due to normal function of the other vas deferens.^5^

Cystic fibrosis transmembrane conductance regulator (*CFTR*) variants are responsible for cystic fibrosis (CF) and were found to play a crucial role in the development of CUAVD.^6^ Most CAVD patients are compound heterozygotes with different mutant alleles, and about 43% of CUAVD patients carry at least one *CFTR* variant according to previous reports.^7^ Among these genotypes/alleles, 5T, F508del, and R117H are the most common. These variants exhibit striking ethnic discrepancies, with a higher frequency in Caucasians associated with the CAVD phenotype than in non-Caucasians.^1^ While the 5T variant is often considered a mild variant,^8^ in combination with (TG)12 or (TG)13 repeats in *CFTR* intron 8, it may enhance CAVD severity.^9^

Recently, a meta-analysis summarized the *CFTR* variant profiles of CBAVD patients, with 78% of patients having at least one *CFTR* variant, 46% having two, and 28% having exactly one.^10^ Due to statistical errors, another review recalculated these percentages based on the original data cited in the meta-analysis,^6^ resulting in rectified percentages of 53% for two variants and 25% for one variant. Several studies have also reported *CFTR* variants in non-CAVD infertile patients,^11,12^ indicating that those with *CFTR* variants in general are at higher risk of infertility even without CAVD.

Renal abnormality (RA) is sometimes detected during CAVD assessment.^7,13,14^ Moreover, although several studies have reported *CFTR* variants in CAVD patients with or without RA,^7,15^ others have found no *CFTR* variants among individuals with accompanying RA.^13,16^ Therefore, the general incidence of RA among CAVD patients and the relationship between CAVD-associated RA and *CFTR* variants remain unknown.

Thus far, results from previous studies on the link between *CFTR* variants and CUAVD have disagreed. Ethnic differences, variation in scanning methods, or case heterogeneity may account for these discrepancies. However, to date, there are no systematic reviews that summarize the general profile of *CFTR* variants among CUAVD patients. Therefore, in this study, we aimed to perform a systematic review and meta-analysis to elucidate the general profile of *CFTR* variants among CUAVD patients, as well as the relationship between *CFTR* variants and CUAVD-associated RA.

## Materials and methods

### Literature search strategy

This systematic review, including the literature search strategy, study selection, and summary of results, was conducted in accordance with Preferred Reporting Items for Systematic Reviews and Meta-Analyses (PRISMA) guidelines.^17^ Literature search was limited to 31 May 2017 and conducted using electronic databases, including PubMed, MEDLINE, Embase, and Cochrane Libraries, to identify studies related to *CFTR* variant and CUAVD. The following search terms were used without language restrictions: “congenital unilateral absence of the vas deferens,” “CUAVD,” “CAVD,” “cystic fibrosis transmembrane conductance regulator,” “CFTR,” “mutation,” “variant,” “frequency,” “genotype,” “allele,” “F508del,” “5T,” and “R117H” (Supplementary Table S1). Additionally, relevant studies from the references of all retrieved publications and review articles were manually identified and included. This study was approved by the Institutional Review Board of the Family Planning Research Institute, Tongji Medical College, Huazhong University of Science and Technology (Wuhan, China).

### Study selection and eligibility criteria

Two reviewers (HCC and XJS) performed an initial screening of all titles and abstracts independently. Studies were considered eligible if they (1) reported *CFTR* variants in CUAVD cases; (2) described genotyping protocols; (3) diagnosed CUAVD using a comprehensive strategy including physical examination, semen analysis, and transrectal ultrasound; (4) reported the case frequency of *CFTR* variant, common genotypes/alleles as primary outcomes, and RA frequency as a secondary outcome (at least one primary indicator was involved in a single study); and (5) were observational (case control or cross-sectional) studies. Studies of poor quality, such as those with ambiguous inclusion/exclusion criteria, genotyping protocols, or diagnostic information, were excluded. Review articles, conference abstracts, unpublished data, and case reports were considered ineligible. Any discrepancy was resolved by consensus among all authors.

### Data extraction and methodological quality evaluation

Basic information was extracted from the eligible studies by two authors independently. CUAVD patients with typical CF symptoms or RA were considered ineligible for calculating the pooled effect size of *CFTR* variants, because such cases may represent a distinct clinical entity with different genetic etiology from isolated CUAVD.^18^ To avoid the inclusion of duplicate or overlapping samples, we meticulously compared the original areas of the studies and details of the author affiliations and included the latest version of each data set with the largest number of cases or adjusted odds ratios (ORs) and 95% confidence intervals (CIs).^19^

### Data synthesis and meta-analysis

For single proportions, we calculated prevalence estimates using the variance-stabilizing Freeman–Tukey double arcsine transformation.^20^ Meta-analyses were carried out after normality tests (*P* > 0.05). Combined effect ORs and 95% CIs were calculated with the Mantel–Haenszel method as the main outcomes. Study heterogeneity was assessed using a *Q* test and the *I*^*2*^ index. A fixed-effect model was applied in the presence of mild or no heterogeneity, while a random-effect model was used when significant heterogeneity was present. Subgroup analyses were performed according to differences in ethnicity or regional origin, presence of typical CF and RA, genotyping method (whole exon sequencing or common variant screening), and study type (case control study or cross-sectional study) to identify substantial heterogeneity. Comparisons among subgroups were analyzed by chi-square (χ^2^) test. A two-sided *P* value ≤0.05 was considered significant.

### Sensitivity analyses and publication bias assessment

Sensitivity analyses were performed when substantial heterogeneity was detected. The existence of publication bias was assessed with funnel plots and Begg’s and Egger’s tests.^21,22^ When asymmetric funnel plots were observed, contour-enhanced funnel plots using the trim-and-fill method were adopted to further help identify publication bias and other causes of asymmetry.^23^

### Analysis software

The meta-analysis and construction of forest and funnel plots were performed with R Software (R version 3.4.0, R package for meta-analysis). GraphPad Prism (version 6.0c; GraphPad Software, San Diego, CA, USA) and SPSS Statistics (version 23.0; IBM, Armonk, NY, USA) were utilized for statistical analysis.

## Results

### Identification of literature

**Fig. 1:**
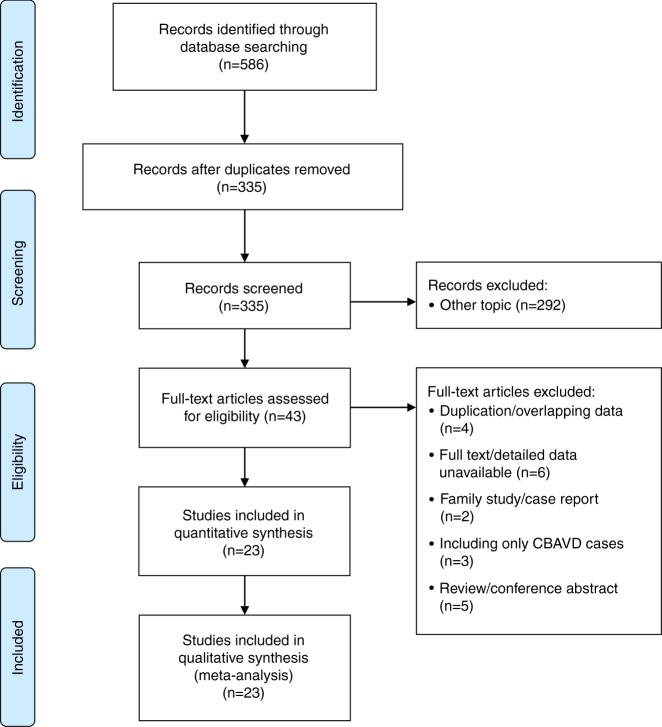
**Schematic of study selection**

A total of 586 articles were identified through a comprehensive literature search of the main databases, 251 of which were excluded as duplicates. The remaining 335 records were subsequently screened based on their titles and abstracts. The search strategy is provided in Supplementary Table S1. Overall, 43 of the identified articles were reviewed in full for eligible data, and 20 were ultimately excluded for reasons listed in Supplementary Table S2. Finally, 23 studies met the inclusion criteria and were included in the quantitative synthesis. Fig. 1 provides an overview of the process of literature searching, screening, and systematic review.

### Characteristics of included studies and quality assessment

In total, 23 studies^1,7,13,14,16,24–41^ provided *CFTR* variant profiles in 141 CUAVD cases. Specific testing for 5T was also performed in all except three studies^7,16,41^ (Supplementary Table S3). However, only 5 studies^1,14,26–28^ determined the CUAVD risk for the allele 5T, where normal fertile males or non-CAVD infertile patients were selected as controls. Some studies involved CUAVD cases with minor CF-related symptoms, such as respiratory tract symptoms or pancreatitis episodes.^1,26,35^ As demonstrated in Table 1, among the 23 studies, 15 studies^7,13,14,16,24–26,28–31,33,36–38^ included CUAVD cases with RA; RA status was unclear in the remaining eight studies, and therefore the relevant data from these studies were further excluded before conducting the RA analysis. The eligible studies were heterogeneous in terms of subject ethnicity because the studies were conducted in a wide range of populations. A few studies even included individuals from different geographical locations/ethnic origins. Most studies employed a comprehensive strategy for variant detection, with 12 studies screening the full sequences of 27 exons and flanking regions, and 11 detecting all or most of the common *CFTR* variants or several specific ones. Characteristics of the included studies are provided in detail in Table 1.

**Table 1:** Characteristics of the included studies

Author	Country /ethnicity	CAVD diagnosis	Genotyping method	Study type	Case of RA (event/total)		Included cases		Control
					CUAVD	CBAVD	CUAVD	CBAVD	
Yang et al. (2015)^24^	Chinese	CSC; excluding CF	Whole exon/flanking sequence	Case control	1/6	1/11	5	10	50 NC
Chiang et al. (2013)^25^	Taiwanese	CSC; excluding CF and RA;	Whole exon/flanking sequence	Cross-sectional	1/2	0/12	1	12	NA
Schwarzer and Schwarz (2012)^13^	German	CSC; excluding CF and RA	Common variants screening	Cross-sectional	5/13	5/110	8	105	NA
Sharma et al. (2009)^26^	Indian	CSC; excluding CF and CFAS and RA	Whole exon/flanking sequence; 5T and TG test	Case control	3/10	1/40	7	39	50 NC
Radpour et al. (2007)^27^	Iranian	CSC; excluding CF and RA	Whole exon/flanking sequence; 5T and TG test	Case control	NA	0/112	7	112	84 NC
Danziger et al. (2004)^29^	U: Hispanic B: nine Asian or Asian-Indian, three Caucasian, and one Hispanic	CSC; excluding CF and RA	Common variants screening	Cross-sectional	0/1	2/13	1	11	2 OA
Grangeia et al. (2004)^28^	Portuguese	CSC; excluding CF and RA	Whole exon/flanking sequence; 5T test	Case control	0/4	3/34	4	31	114 NC; 16 OAZ and 23 NOAZ
Kolettis and Sandlow (2002)^31^	American	CSC; excluding CF and RA	Common variants screening	Cross-sectional	4/11	NA	6	NA	NA
Robert et al. (2002)^30^	French	CSC; excluding CF and RA	Common variants screening; 5T test	Cross-sectional	1/7	2/40	6	38	NA
Attardo et al. (2001)^33^	Italian	CSC; excluding CF and RA	Common variants screening; 5T test	Cross-sectional	0/1	3/37	1	34	NA
Larriba et al. (2001)^32^	Spanish	CSC; excluding CF	Whole exon/flanking sequence 5T test	Cross-sectional	NA	NA	4	16	30 Non-CAVD
Casals et al. (2000)^14^	Spanish	CSC; excluding CF and RA	Whole exon/flanking sequence; 5T test	Case control	10/24	6/110	14	104	200 NC
Jézéquel et al. (2000)^35^	French	CSC; excluding CF and CFAS; RAU	Whole exon/flanking sequence; 5T test	Cross-sectional	NA	NA	3	37	7 OA
Zeng et al. (2000)^34^	Chinese	CSC	Common variants screening	Cross-sectional	NA	NA	15	NA	NA
Castellani et al. (1999)^36^	Italian	CSC; excluding CF and RA	Common variants screening; 5T test	Cross-sectional	0/3	2/39	3	37	NA
Boucher et al. (1999)^37^	French	CSC; excluding CF and RA	Common variants screening	Cross-sectional	0/2	1/12	2	11	39 Non-CAVD; 37 oligozoospermia
Dörk et al. (1997)^38^	German with a few Austrian, Portuguese, Turkish, and Vietnamese	CSC; excluding CF and RA	Whole exon/flanking sequence; 5T test	Cross-sectional	0/5	9/101	5	92	NA
Schlegel et al. (1996)^16^	American	CSC; excluding CF and RA	Common variants screening	Cross-sectional	5/19	6/53	12	52	NA
Casals et al. (1995)^40^	Spanish	CSC; excluding CF	Common variants screening	Cross-sectional	NA	NA	6	28	NA
Chillón et al. (1995)^1^	European (Belgian, French, Spanish) and American	CSC; excluding CF and CFAS; RAU	Whole exon/flanking sequence; 5T test	Case control	NA	NA	12	102	46 NC 10 non-CAVD
Jarvi et al. (1995)^39^	Canadian	CSC; excluding CF	Common variants screening; 5T test	Cross-sectional	NA	NA	2	25	17 OAZ; 18 SF
Mickle et al. (1995)^7^	American	CSC; excluding CF and RA	Common variants screening	Cross-sectional	5/21	NA	16	NA	NA
Culard et al. (1994)^41^	French including one Turkish	CSC; excluding CF; RAU	Whole exon/flanking sequence	Cross-sectional	NA	NA	1	8	NA

*CAVD* congenital absence of the vas deferens, *CBAVD* congenital bilateral absence of the vas deferens, *CF* cystic fibrosis, *CFAS* CF-related atypical symptoms that comprise mainly minor pulmonary and gastrointestinal, *CSC* CUAVD diagnosis conformed to the standardized criteria, *CUAVD* congenital unilateral absence of the vas deferens, *NA* not available, *NC* normal controls, *NOAZ* nonobstructive azoospermia, *non-CAVD* noncongenital absence of the vas deferens infertility, *OAZ* obstructive azoospermia, *RA* renal abnormalities that include mainly uni-/bilateral renal agenesis, *RAU* renal abnormality information was unavailable, *SF* spermatogenic failure

### Summary frequencies of *CFTR* variants in CUAVD patients

In all, 23 eligible studies provided sufficient data for summary analysis of the overall frequency of *CFTR* variants among CUAVD patients (Supplementary Table S4). The results showed that 46% of patients with CUAVD had at least one *CFTR* variant (*I*^2^ = 53%, *P* < 0.01), with 27% having one (*I*^2^ = 54%, *P* < 0.01) and only 5% having two (*I*^2^ = 39%, *P* = 0.03), all with moderate heterogeneity. Publication bias was evident in those having at least one (*P* = 0.010) and those having exactly one (*P* = 0.018) *CFTR* variant, while no publication bias was detected in those having two variants (*P* = 0.148), as demonstrated by funnel plots and Egger’s test (Supplementary Figs. S1–S3 and S9–S11).

Summary analysis demonstrated that the frequency of F508del/5T, the most common heterozygous genotype previously reported among CBAVD patients,^10^ was 0% (95% CI = 0–2%) among CUAVD patients (Supplementary Fig. S4). As for frequent mutant alleles, the frequency of F508del was 4%, and that of 5T was 9% (Supplementary Figs. S5 and S6). Heterogeneity was not significant for any common genotype/allele except 5T (*I*^2^ = 44%, *P* = 0.02, Supplementary Table S4). Moreover, publication bias was evident for all but the common variants of F508del and 5T, as demonstrated by Egger’s test (*P* = 0.117 and *P* = 0.136, respectively, Supplementary Table S4) and symmetric funnel plots (Supplementary Figs. S12–S14). We also calculated the pooled frequencies of the common F508del/R117H genotype and R117H allele, both of which were 0% among CUAVD patients (Supplementary Figs. S7, S8, S15, and S16).

Furthermore, we employed contour-enhanced funnel plots using the trim-and-fill method to detect the causes of funnel plot asymmetry. Publication bias was determined to be responsible for the presence of asymmetry in a funnel plot if the “missing” studies were distributed across the insignificant area, while other factors were determined to account for the asymmetry if the “filled” studies were scattered in the significant areas.^23^ As a result, we found that publication bias accounted for all instances of asymmetry except for in the case of the pooled *CFTR* frequency among CUAVD patients with two variants, indicating that other factors may be involved (Supplementary Fig. S17A–E).

### Subgroup analyses of *CFTR* variants in CUAVD patients

**Fig. 2:**
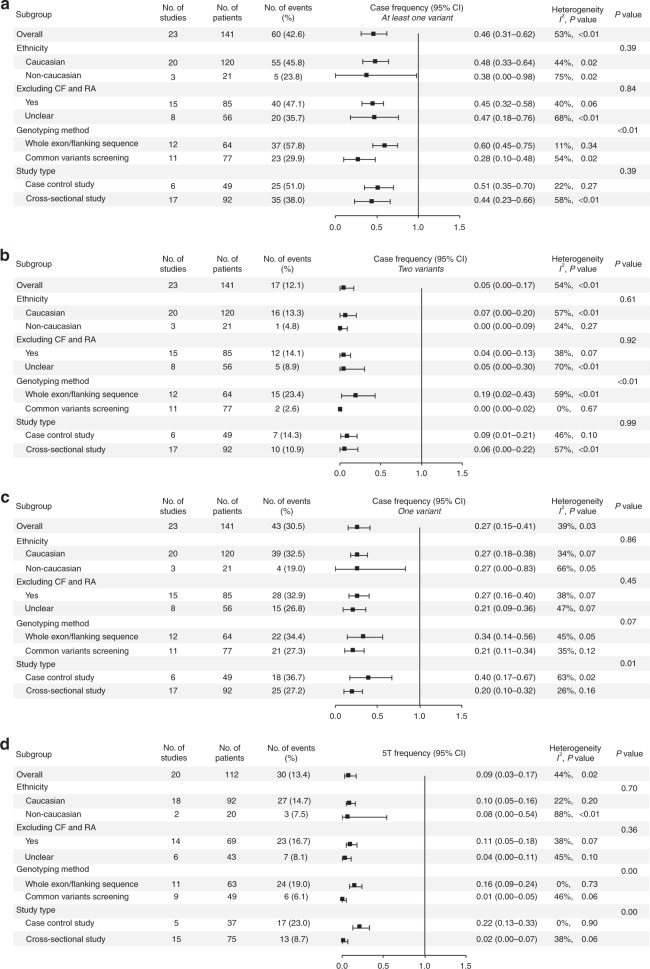
**Subgroup analysis of** ***CFTR*** **variants and 5T allele frequency in patients with congenital unilateral absence of the vas deferens (CUAVD).** Solid squares indicate the pooled effect size of each study, with horizontal lines representing the 95% confidence interval (CI). **a** Frequency of at least one *CFTR* variant. **b** Frequency of two *CFTR* variants. **c** Frequency of one *CFTR* variant. **d** Frequency of 5T allele. *CF* cystic fibrosis, *RA* renal abnormalities.

As mentioned above, except for the four common genotypes/alleles of F508del/5T, F508del, F508del/R117H, and R117H (*I*^2^ < 30%, *P* > 0.05), heterogeneity was significant for all other outcomes (genotypes and alleles) (*I*^2^ > 30%, *P* < 0.05). Subjects from each study were classified into Caucasian and non-Caucasian groups according to the ethnicity/country of origin of the study subjects. Typical CF and RA information was identified throughout the full publication until clear or unclear outcomes were determined. Genotyping methods were generally categorized as whole exon/flanking sequencing or common variant screening based on the information provided in the methods. Additionally, studies were separated into case control or cross-sectional study types. The results of all subgroup analyses are presented in Fig. 2. Based on these results, we determined that heterogeneity in the 5T allele might be attributed to genotyping method and study type (Fig. 2d), whereas other factors only minimally explained the sources of heterogeneity.

Comparisons among subgroups demonstrated that, compared with common variant screening, whole exon/flanking sequencing resulted in a higher frequency of cases with at least one or two *CFTR* variants (at least one variant: 60% vs. 28%, *P* = 0.0002; two variants: 19% vs. 0%, *P* < 0.0001, respectively, Fig. 2a, b), as well as a higher frequency of cases with the 5T allele (16% vs. 1%, *P* = 0.0024, respectively, Fig. 2d). For case control and cross-sectional studies, the frequencies of cases with only one variant were 40% and 20%, respectively (*P* = 0.0068, Fig. 2d), while the frequencies of those with the 5T allele were 22% and 2%, respectively (*P* = 0.0029, Fig. 2d). Apart from these, there were no significant differences between other subgroups in terms of pooled case frequencies or common genotype/allele frequencies. The results of the sensitivity analysis (Supplementary Figs. S18–S25) further supported our conclusions.

### Pooled OR of 5T allele for CUAVD risk

**Fig. 3:**
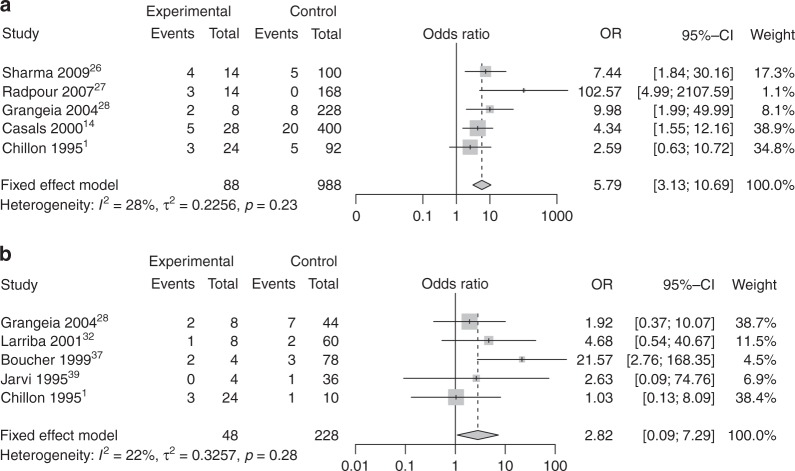
**Forest plots for meta-analysis of pooled odds ratio (OR) of 5T allele in patients with congenital unilateral absence of the vas deferens (CUAVD).** Summary ORs and their 95% confidence intervals (CIs) were calculated by Mantel–Haenszel method and are indicated with diamonds. Solid squares indicate the OR of each study, with the square size directly proportional to the weight and horizontal lines representing 95% CIs. Dotted vertical line indicates the overall estimate, and solid black line indicates the null effect (OR = 1). **a** CUAVD vs. normal controls. **b** CUAVD vs. non-CAVD infertile males

Overall, five studies^1,14,26–28^ comprising 88 CUAVD patients and 988 normal controls, and another five studies^1,28,32,37,39^ comprising 48 CUAVD patients and 228 non-CAVD patients, were eligible for meta-analysis of the OR of the 5T allele. Under a fixed-effect model, the pooled OR for 5T among CUAVD patients was 5.79 (95% CI = 3.13–10.69, *I*^2^ = 28%, *τ*^2^ = 0.23, *P* < 0.0001, Fig. 3a) compared with normal controls and 2.82 (95% CI = 1.09–7.29, *I*^2^ = 22%, *τ*^2^ = 0.33, *P* = 0.032, Fig. 3b) compared with non-CAVD males. For both, the heterogeneity among studies was mild. In the first analysis, the 5T frequency in CUAVD patients was 19.3% compared with 3.8% among normal controls, with an absolute difference of 15.5%, while in the second analysis, it was 16.7% in CUAVD patients compared with 6.1% in non-CAVD males, with an absolute difference of 10.5% (Supplementary Table S3).

### Summary frequency of RA in CAVD patients

**Fig. 4:**
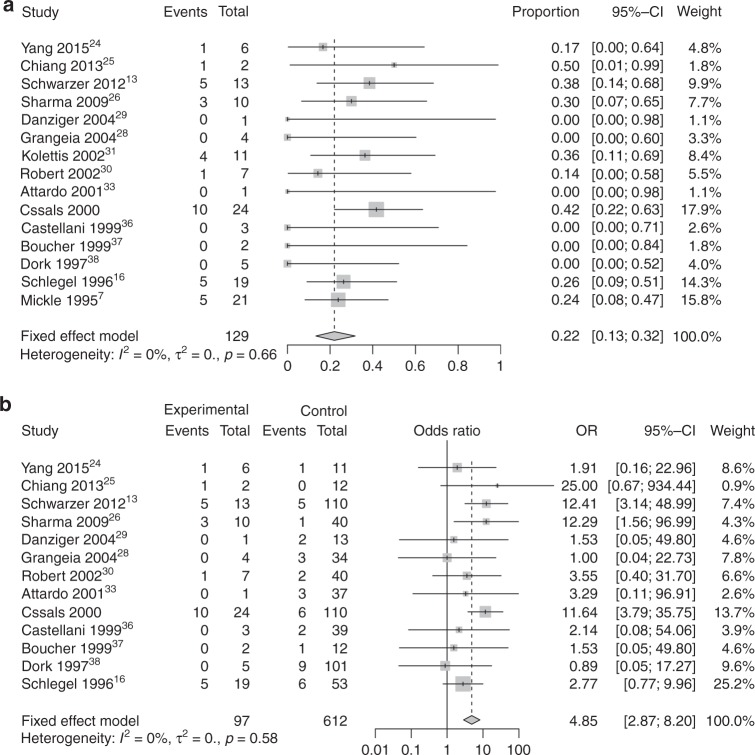
**Forest plots for meta-analysis of renal abnormality (RA) frequency and pooled odds ratio (OR) of RA risk in patients with congenital unilateral absence of the vas deferens (CUAVD).** See details in Fig. 3. **a** RA frequency in CUAVD patients. **b** Pooled OR of RA risk in CUAVD patients

Among the 23 studies, 15 reported RA information for CUAVD patients, while 14 reported RA information for CBAVD patients. The results of the resulting meta-analysis and subgroup analysis are summarized in Fig. 4 and Supplementary Table S3.

Summary analysis showed that 22% of CUAVD patients had RA (Fig. 4a), with no heterogeneity (*I*^2^ = 0%, *P* = 0.66) but evident publication bias as demonstrated by funnel plots and Egger’s test (*P* = 0.03, Supplementary Fig. S26). A contour-enhanced funnel plot indicated that publication bias failed to explain the origin of the funnel plot asymmetry (Supplementary Fig. S17F). The actual RA frequency in CUAVD patients was 26.8% (35/129) compared with 6.7% (49/724) in CBAVD patients, with an absolute difference of 20.1% (Supplementary Table S3). Among the 35 CUAVD patients with accompanying RA, only 8.6% (3/35) of the cases in one study^14^ were found to carry one *CFTR* variant (with one patient carrying each of the F508del, 3732delA, and 5T variants), while 45.3% (39/86, with eight patients declining to undergo *CFTR* screening) of those without RA were positive for *CFTR* variants, indicating a low possibility that *CFTR* gene variants were associated with RA among CUAVD patients (Supplementary Table S5).

Among CBAVD patients, only 5% had accompanying RA (Supplementary Fig. S27), a value that was significantly lower than that of CUAVD patients (5% vs. 22%, *P* < 0.0001, χ^2^ = 44.17), with moderate heterogeneity (*I*^2^ = 52%, *P* = 0.01) and no publication bias as demonstrated by funnel plots and Egger’s test (*P* = 0.17, Supplementary Fig. S28). Furthermore, subgroup analysis for RA in CBAVD patients, including stratification by ethnicity, genotyping method, and study type, was performed to explore sources of heterogeneity. However, none of the factors explained the origin of the heterogeneity (Supplementary Fig. S29). Sensitivity analyses were also conducted and further supported our conclusions (Supplementary Figs. S30 and S31).

### Pooled OR of RA risk in CAVD patients

Among the 15 studies mentioned above, 13 studies provided RA information for CUAVD and CBAVD patients at the same time (two studies^7,31^ did not provide RA information for CBAVD patients and were excluded), including 97 CUAVD and 612 CBAVD patients. Under a fixed-effect model, the pooled OR of RA risk among CUAVD patients was 4.85 (95% CI = 2.87–8.20, *I*^2^ = 0%, *τ*^*2*^ = 0, *P* < 0.0001, Fig. 4) compared with CBAVD, with no heterogeneity or publication bias (*P* = 0.06, Supplementary Fig. S32). Sensitivity analysis also supported this conclusion (Supplementary Fig. S33).

## DISCUSSION

### Principal findings

In the present study, we discovered a fairly high frequency of overall *CFTR* variants in CUAVD patients. However, the frequencies of the heterozygous genotypes F508del/5T and F508del/R117H were very low. Additionally, CUAVD patients had an increased 5T risk allele frequency of 15.5% compared with that in normal controls, but an increase of only 10.5% compared with that in non-CAVD males. The number needed to harm (NNH) of the 5T risk was six for CUAVD patients, i.e., for every six CUAVD patients, one more 5T variant should occur. In contrast, the number of non-CAVD males required to add one more 5T case was 10. Subgroup analysis revealed that genotyping method and study type might contribute to the heterogeneity in 5T variant frequencies, irrespective of the influence of ethnicity. This is quite different from CBAVD, in which ethnicity plays an important role.^6^ Interestingly, we also discovered that CUAVD patients bear a higher level of RA risk than CBAVD patients, with an absolute risk increase of 20.1%. In this case, the NNH was five, i.e., for every five CUAVD patients, one more RA case should occur. Moreover, *CFTR* variants appeared to have little relationship with CUAVD-associated RA.

### Interpretation of the findings

Compared with the *CFTR* variant profile in CBAVD patients, as reported in a study by Yu et al.,^10^ the profile in CUAVD is considerably different. Reduced frequencies of cases and common heterozygous genotypes/alleles were observed in general, suggesting that a gene dosage effect may be involved.^1^ Most patients with two *CFTR* variants possessed a heterozygous genotype, with one severe variant commonly present with a mild allele.^42^ To some extent, this may explain the lower frequency of the severe *CFTR* variant F508del in CUAVD patients, as CUAVD could be an incomplete form of CBAVD.^43^ The frequency of common heterozygous genotypes in CBAVD, for instance F508del/5T and F508del/R117H, however, was very low in CUAVD. Moreover, F508del, one of the most common and severe variants in CF, was observed in around 17% of CBAVD patients, while the milder 5T allele was observed in about 25% (ref. ^6^). The corresponding frequencies in CUAVD patients were 4% and 9%, respectively, based on the current study. The F508del variant is the most common variant associated with CF in Caucasians and impairs CFTR protein folding and trafficking.^44^ Our results are consistent with the assumption that severe variants such as F508del would result in typical CF, while the mild variant 5T might be responsible for atypical CF symptoms, such as CBAVD and CUAVD.^45^

A polymorphic variant in *CFTR* intron 8, 5T causes less efficient exon 9 splicing and reduced expression of functional CFTR protein. It is considered to be a pathogenic variant linked to CBAVD or other atypical symptoms of CF.^46^ Moreover, the 5T variant in combination with longer (TG)12 or (TG)13 repeats probably results in an increased disease risk compared with that of 5T itself.^47^ In vitro studies have shown that 5T/(TG)12 results in shorter transcript variants, leading to deficient CFTR protein function and consequently inducing abnormal fluid secretion and electrolytes.^48^ However, owing to the limited data extracted from the original studies, we did not conduct a meta-analysis of 5T/(TG)12_13 frequency here. Yet, our summary ORs indicate an increased CUAVD risk for males carrying the 5T allele compared with those of normal controls and non-CAVD males. Therefore, more studies are needed to further elucidate the effects and molecular mechanisms of different combinations of 5T and TG repeats.

The R117H variant is reported to affect CFTR channel conductivity without influencing the quantity or structure of CFTR proteins.^49^ Unlike the F508del variant, R117H is thought to induce mild effects, producing less severe clinical symptoms, such as CBAVD or CUAVD.^50^ The frequency of R117H variant was found to be approximately 0% among CUAVD patients in the current study compared with 3% among CBAVD patients according to a previous study.^10^ This may indicate that R117H variant is not the main predictor of CUAVD. However, due to the limited number of patients included, more studies are needed to confirm our conclusions.

Current evidence emphasizes the pivotal role of ethnicity in the *CFTR* variant profile of CAVD patients. Results from several studies have shown that *CFTR* variant is common among Caucasians with CBAVD^32,35^ and very rare in non-Caucasians;^24,25^ this is especially true for the common variants F508del, 5T, and R117H. However, this racial discrepancy was not statistically significant among CUAVD patients in the present study, which may be attributed to the small sample size of non-Caucasians, with only two or three studies included. Even though some of the studies themselves included patients from various countries or regions, in these cases, the data could not be extracted for subgroup analysis, thus increasing heterogeneity among the studies. Therefore, large-scale studies among non-Caucasians are needed to further confirm this racial discrepancy.

Undoubtedly, whole exon sequencing offers an advantage over common variant screening in detecting rare variants. According to subgroup analyses, a higher case frequency of *CFTR* variant was observed when using whole exon sequencing rather than common variant screening. Because of this, researchers have recently proposed that whole exon sequencing be used in both OA and nonobstructive azoospermia before intracytoplasmic sperm injection.^6,51^ Due to the use of multiple genotyping methods in the included studies, the real incidence of *CFTR* variant in CUAVD patients may be underestimated.

In 1737, John Hunter first described CAVD in a cadaver.^52^ An association with renal agenesis has subsequently been noted.^4,14,53^ We observed a high RA risk in CUAVD patients of 22%, which is around five times higher than that in CBAVD (5%). No *CFTR* variants were observed in these patients except in one study (with 3/10 CUAVD patients with RA carrying one *CFTR* variant),^14^ in agreement with previous studies as a whole. Relevant studies have suggested that CUAVD accompanied by RA is a special condition with a genetic background distinct from that of typical CUAVD.^38^ In fact, RA-associated CUAVD is due to an intrinsic defect in the Wolffian duct, as any defect or interruption before the complete separation of the Wolffian duct can lead to CUAVD combined with RA, whereas interruption after separation leads to isolated CUAVD.^52^ Our conclusions further support these findings.

### Strengths and limitations

To the best of our knowledge, this is the first systematic review and meta-analysis concerning the overall *CFTR* variant profile and RA risk among CUAVD patients. In our study, a comprehensive search was performed with meticulous search strategies and strict eligibility criteria for the relevant literature. When synthesizing the data for the frequency of *CFTR* variant, CUAVD cases with accompanying RA were excluded, as their genetic origins are different from those of isolated CUAVD.^54^ Moreover, contour-enhanced funnel plots were constructed with the trim-and-fill method when funnel plot asymmetry was observed, to detect sources of bias.^23^ Subgroup and sensitivity analyses were performed to investigate substantial heterogeneity and guarantee the consistency and accuracy of our conclusions. Overall, every effort was made to reduce the risk of bias and heterogeneity to ensure the high quality of the study.

Nevertheless, there remain several drawbacks to our study. First, a limited number of CUAVD patients were included in this study, which may lead to substantial bias in outcomes. Second, confounding factors such as multiple ethnicities, genotyping methods, unclear renal conditions, and small sample sizes, may affect outcomes. Moreover, the existence of publication bias, as validated by contour-enhanced funnel plots and Egger’s test, potentially increases the risk of overestimation and thus overdiagnosis and overtreatment. Lastly, the eligible observational studies themselves are a potential source of bias, although previous studies have confirmed the role of meta-analysis in incorporating observational studies.^55^ Hence, special caution should be taken when considering these results.

### Implications for clinical practice and future research

With the help of assisted reproductive technology (ART), CUAVD infertile males bearing *CFTR* variants can become biological fathers. Consequently, detrimental variants may be transmitted vertically to offspring. More importantly, given the evidence that *CFTR* variants may affect sperm production, maturation, and fertilization,^6^ it is necessary for these individuals to turn to genetic counseling before undergoing ART to comprehensively assess the genetic risk for their progeny. Currently, there is no consensus method for *CFTR* variant detection in clinical settings, thus neglecting less frequent variants.

Furthermore, delayed diagnosis of CUAVD can increase mortality and morbidity due to associated defects of the urogenital system.^52^ Given the high frequency of RA risk in CUAVD, imaging of the urogenital system is strongly recommended in clinical practice to better evaluate patient general health and quality of life.

In conclusion, CUAVD patients exhibit a fairly high frequency of *CFTR* variants, with 5T and F508del being the most common. CUAVD patients bear a higher RA risk than CBAVD patients, although no relationship was detected between CUAVD-associated RA and *CFTR* variants. Whole exon/flanking sequencing of *CFTR* and renal ultrasound examination are recommended when consulting with CUAVD patients in the clinic.

## Electronic supplementary material

Supplementary Figures

Supplementary Tables
