# Supplementary material for: CFTR variants and renal abnormalities in males with congenital unilateral absence of the vas deferens (CUAVD): a systematic review and meta-analysis of observational studies
Source: Genet Med. 2018 Sep 14;21(4):826–36. doi: 10.1038/s41436-018-0262-7 (PMC6752674; doi:10.1038/s41436-018-0262-7)
Supplement: Supplementary file 1 — Supplementary Figures [file 41436_2018_262_MOESM1_ESM.docx]

**Supplemental Figures**

**Figure Legends**

**Supplementary Figure S1** Forest plot for meta-analysis of *CFTR* frequency with at least one variant in CUAVD patients. Summary frequency of CUAVD cases with at least one variant, and their 95% CI were calculated by random-effect model. Summary frequency and 95% CI are indicated by diamond. Solid grey square marks the frequency from each study with square size directly proportional to the weight, and the horizontal lines represent 95% CI.

**Supplementary Figure S2** Forest plot for meta-analysis of *CFTR* frequency with two variants in CUAVD patients. For details see **Fig. S1**.

**Supplementary Figure S3** Forest plot for meta-analysis of *CFTR* frequency with one variant in CUAVD patients. For details see **Fig. S1**.

**Supplementary Figure S4** Forest plot for meta-analysis of F508del/5T frequency in CUAVD patients. For details see **Fig. S1**.

**Supplementary Figure S5** Forest plot for meta-analysis of F508del frequency in CUAVD patients. For details see **Fig. S1**.

**Supplementary Figure S6** Forest plot for meta-analysis of 5T frequency in CUAVD patients. For details see **Fig. S1**.

**Supplementary Figure S7** Forest plot for meta-analysis of F508del/R117H frequency in CUAVD patients. For details see **Fig. S1**.

**Supplementary Figure S8** Forest plot for meta-analysis of R117H frequency in CUAVD patients. For details see **Fig. S1**.

**Supplementary Figure S9** Funnel plots for meta-analysis of *CFTR* frequency with at least one variant in CUAVD patients.

**Supplementary Figure S10** Funnel plots for meta-analysis of *CFTR* frequency with two variants in CUAVD patients.

**Supplementary Figure S11** Funnel plots for meta-analysis of *CFTR* frequency with one variant in CUAVD patients.

**Supplementary Figure S12** Funnel plots for meta-analysis of F508del/5T frequency in CUAVD patients.

**Supplementary Figure S13** Funnel plots for meta-analysis of F508del frequency in CUAVD patients.

**Supplementary Figure S14** Funnel plots for meta-analysis of 5T frequency in CUAVD patients.

**Supplementary Figure S15** Funnel plots for meta-analysis of F508del/R117H frequency in CUAVD patients.

**Supplementary Figure S16** Funnel plot for meta-analysis of R117H frequency in CUAVD patients.

**Supplementary Figure S17** Contour-enhanced funnel plots with trim-and-fill method for meta-analysis of *CFTR* variant and RA frequency in CUAVD. The open circle marks the “filled” studies, while solid circle represents the primary studies. A. At least one variant; B. Two variants; C. F508del/5T genotype; D. F508del/R117H genotype; E. R117H allele; F. Renal abnormality (RA).

**Supplementary Figure S18** Sensitivity analysis plot for meta-analysis of *CFTR* frequency with at least one variant in CUAVD patients.

**Supplementary Figure S19** Sensitivity analysis plot for meta-analysis of *CFTR* frequency with two variants in CUAVD patients.

**Supplementary Figure S20** Sensitivity analysis plot for meta-analysis of *CFTR* frequency with one variant in CUAVD patients.

**Supplementary Figure S21** Sensitivity analysis plot for meta-analysis of F508del/5T frequency in CUAVD patients.

**Supplementary Figure S22** Sensitivity analysis plot for meta-analysis of F508del frequency in CUAVD patients.

**Supplementary Figure S23** Sensitivity analysis plot for meta-analysis of 5T frequency in CUAVD patients.

**Supplementary Figure S24** Sensitivity analysis plot for meta-analysis of F508del/R117H frequency in CUAVD patients.

**Supplementary Figure S25** Sensitivity analysis plot for meta-analysis of R117H frequency in CUAVD patients.

**Supplementary Figure S26** Funnel plot for meta-analysis of RA frequency in CUAVD patients.

**Supplementary Figure S27** Forest plots of meta-analysis for RA frequency in CBAVD.

**Supplementary Figure S28** Funnel plot for meta-analysis of RA frequency in CBAVD patients.

**Supplementary Figure S29** Subgroup analyses for meta-analysis of RA frequency in CBAVD patients.

**Supplementary Figure S30** Sensitivity analysis plot for meta-analysis of RA frequency in CUAVD patients.

**Supplementary Figure S31** Sensitivity analysis plot for meta-analysis of RA frequency in CBAVD patients.

**Supplementary Figure S32** Funnel plots for meta-analysis of RA risk in CUAVD patients

**Supplementary Figure S33** Sensitivity analysis plot for meta-analysis of pooling OR for RA risk in CUAVD patients.

**Supplementary Figure S1** Forest plot for meta-analysis of *CFTR* frequency with at least one variant in CUAVD patients. Summary frequency of CUAVD cases with at least one variant, and their 95% CI were calculated by random-effect model. Summary frequency and 95% CI are indicated by diamond. Solid grey square marks the frequency from each study with square size directly proportional to the weight, and the horizontal lines represent 95% CI.

**Supplementary Figure S2** Forest plot for meta-analysis of *CFTR* frequency with two variants in CUAVD patients. For details see **Fig. S1**.

**Supplementary Figure S3** Forest plot for meta-analysis of *CFTR* frequency with one variant in CUAVD patients. For details see **Fig. S1**.

**Supplementary Figure S4** Forest plot for meta-analysis of F508del/5T frequency in CUAVD patients. For details see **Fig. S1**.

**Supplementary Figure S5** Forest plot for meta-analysis of F508del frequency in CUAVD patients. For details see **Fig. S1**.

**Supplementary Figure S6** Forest plot for meta-analysis of 5T frequency in CUAVD patients. For details see **Fig. S1**.

**Supplementary Figure S7** Forest plot for meta-analysis of F508del/R117H frequency in CUAVD patients. For details see **Fig. S1**.

**Supplementary Figure S8** Forest plot for meta-analysis of R117H frequency in CUAVD patients. For details see **Fig. S1**.

**Supplementary Figure S9** Funnel plots for meta-analysis of *CFTR* frequency with at least one variant in CUAVD patients.

**Supplementary Figure S10** Funnel plots for meta-analysis of *CFTR* frequency with two variants in CUAVD patients.

**Supplementary Figure S11** Funnel plots for meta-analysis of *CFTR* frequency with one variant in CUAVD patients.

**Supplementary Figure S12** Funnel plots for meta-analysis of F508del/5T frequency in CUAVD patients.

**Supplementary Figure S13** Funnel plots for meta-analysis of F508del frequency in CUAVD patients.

**Supplementary Figure S14** Funnel plots for meta-analysis of 5T frequency in CUAVD patients.

**Supplementary Figure S15** Funnel plots for meta-analysis of F508del/R117H frequency in CUAVD patients.

**Supplementary Figure S16** Funnel plot for meta-analysis of R117H frequency in CUAVD patients.

**
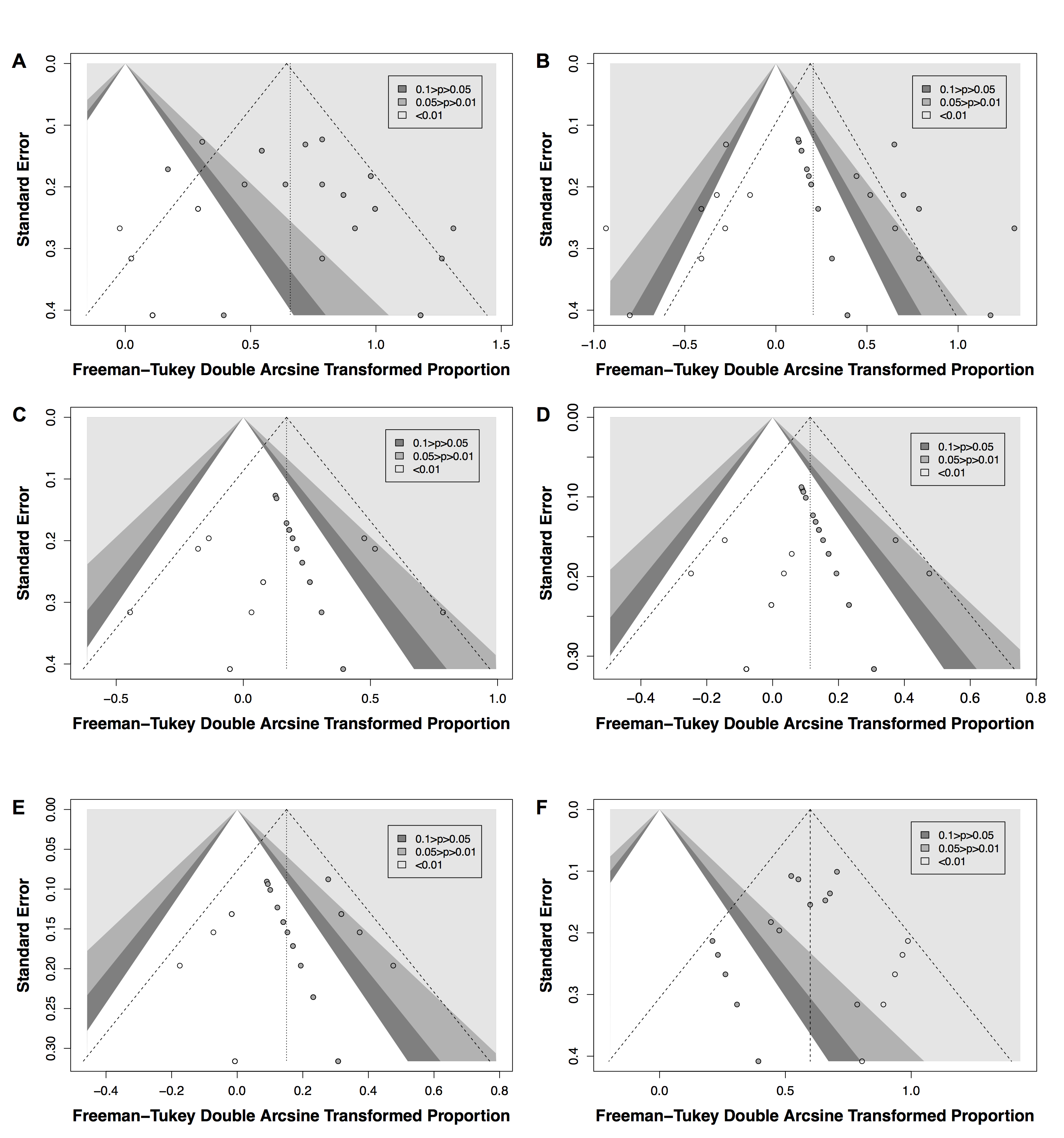
**

**Supplementary Figure S17** Contour-enhanced funnel plots with trim-and-fill method for meta-analysis of *CFTR* variant and RA frequency in CUAVD. The open circle marks the “filled” studies, while solid circle represents the primary studies. A. At least one variant; B. Two variants; C. F508del/5T genotype; D. F508del/R117H genotype; E. R117H allele; F. Renal abnormality (RA).

**Supplementary Figure S18** Sensitivity analysis plot for meta-analysis of *CFTR* frequency with at least one variant in CUAVD patients.

**Supplementary Figure S19** Sensitivity analysis plot for meta-analysis of *CFTR* frequency with two variants in CUAVD patients.

**Supplementary Figure S20** Sensitivity analysis plot for meta-analysis of *CFTR* frequency with one variant in CUAVD patients.

**Supplementary Figure S21** Sensitivity analysis plot for meta-analysis of F508del/5T frequency in CUAVD patients.

**Supplementary Figure S22** Sensitivity analysis plot for meta-analysis of F508del frequency in CUAVD patients.

**Supplementary Figure S23** Sensitivity analysis plot for meta-analysis of 5T frequency in CUAVD patients.

**Supplementary Figure S24** Sensitivity analysis plot for meta-analysis of F508del/R117H frequency in CUAVD patients.

**Supplementary Figure S25** Sensitivity analysis plot for meta-analysis of R117H frequency in CUAVD patients.

**Supplementary Figure S26** Funnel plot for meta-analysis of RA frequency in CUAVD patients.

**Supplementary Figure S27** Forest plots of meta-analysis for RA frequency in CBAVD.

**Supplementary Figure S28** Funnel plot for meta-analysis of RA frequency in CBAVD patients.

**Supplementary Figure S29** Subgroup analyses for meta-analysis of RA frequency in CBAVD patients.

**Supplementary Figure S30** Sensitivity analysis plot for meta-analysis of RA frequency in CUAVD patients.

**Supplementary Figure S31** Sensitivity analysis plot for meta-analysis of RA frequency in CBAVD patients.

**Supplementary Figure S32** Funnel plots for meta-analysis of RA risk in CUAVD patients.

**Supplementary Figure S33** Sensitivity analysis plot for meta-analysis of pooling OR for RA risk in CUAVD patients.
