# Supplementary material for: CFTR variants and renal abnormalities in males with congenital unilateral absence of the vas deferens (CUAVD): a systematic review and meta-analysis of observational studies
Source: Genet Med. 2018 Sep 14;21(4):826–36. doi: 10.1038/s41436-018-0262-7 (PMC6752674; doi:10.1038/s41436-018-0262-7)
Supplement: Supplementary file 2 — Supplementary Tables [file 41436_2018_262_MOESM2_ESM.docx]

**Supplemental Tables**

**Table Legends**

**Supplementary Table S1** Search strategies and outcomes in different electronic databases.

**Supplementary Table S2** List of the excluded studies in the systematic review (N = 20).

**Supplementary Table S3** Data extraction of the included studies (N = 23).

**Supplementary Table S4** Summary for meta-analyses and test of publication bias in CUAVD.

**Supplementary Table S5** *CFTR* variant among CUAVD patients complicated with RA.

**Supplementary Table S1** Search strategies and outcomes in different electronic databases.

| **Database** | **Search strategy** | **Output** |
| --- | --- | --- |
| Pubmed  (www.ncbi.nlm.nih.gov/pubmed) | (congenital unilateral absence of the vas deferens OR CUAVD OR congenital absence of the vas deferens or CAVD OR congenital absence of vas deferens OR congenital aplasia of vas deferens OR absence of vas deferens OR vas deferens) and (cystic fibrosis transmembrane conductance regulator OR CFTR OR F508del OR ΔF508 OR delF508 OR 5T OR R117H OR F508del/5T OR F508del/R117H OR frequency OR mutation(s) OR allele(s) OR genotype OR genogroup(s)) | 434 |
| Ovid MEDLINE  (http://ovidsp.tx.ovid.com)  (1946 to Present with Daily Update) | (congenital unilateral absence of the vas deferens or CUAVD or congenital absence of the vas deferens or CAVD or congenital absence of vas deferens or congenital aplasia of vas deferens or absence of vas deferens or vas deferens) and (cystic fibrosis transmembrane conductance regulator or CFTR or F508del or delF508 or 5T or R117H or F508del/5T or F508del/R117H or frequency or mutation or allele or genotype or genogroup) and (observational study or cohort study or case-control study or cross-sectional study) | 66 |
| EMBASE  (http://ovidsp.tx.ovid.com)  (1974 to 2017 September 13) | #1: congenital unilateral absence of the vas deferens or CUAVD or congenital absence of the vas deferens or CAVD or congenital absence of vas deferens or congenital aplasia of vas deferens or absence of vas deferens or vas deferens (106)  #2: cystic fibrosis transmembrane conductance regulator or CFTR or F508del or delF508 or 5T or R117H or F508del/5T or F508del/R117H or frequency or mutation or allele or genotype or genogroup (2964)  #3: observational study or cohort study or case-control study or cross-sectional study (10389)  #4: #1 and #2 and #3 (73) | 73 |
| Cochrane Library  (http://www.cochranelibrary.com) | #1: CAVD (3)  #2: congenital absence of the vas deferens (6)  #3: congenital absence of vas deferens (6)  #4: congenital aplasia of vas deferens (0)  #5: absence of vas deferens (16)  #6: vas deferens (59)  #7: CFTR (362)  #8: cystic fibrosis transmembrane conductance regulator (191)  #9: F508del (80)  #10: ΔF508 (7)  #11: delF508 (3)  #12: F508del/5T (0)  #13: F508del/R117H (0)  #14: 5T (216)  #15: frequency (53222)  #16: allele (2992)  #17: mutation (s) (4380)  #18: genotype (s) (6786)  #19: genogroup (s) (9)  #20: #1 or #2 or #3 or #4 or #5 or #6 (61)  #21: #7 or #8 or #9 or #10 or #11 or #12 or #13 or #14 or #15 or #16 or #17or #18 or #19 (63320)  #22: #20 and #21 (13) | 13 |
| Total |  | 586 |

**Supplementary Table S2** List of the excluded studies in the systematic review (N = 20).

| **Study ID** | | **Exclusion reason** |
| --- | --- | --- |
| 1. | Akin Y, Demir D, Gorgisen G, Luleci G, Alper OM, Watanabe CS, Sahiner IF, Usta MF. Novel and rare CFTR gene mutations in Turkish patients with congenital aplasia of vas deferens. *Andrologia* 2014;**46**:198-199. | Including only CBAVD cases |
| 2. | Augarten A, Yahav Y, Kerem BS, Halle D, Laufer J, Szeinberg A, Dor J, Mashiach S, Gazit E, Madgar I. Congenital bilateral absence of vas deferens in the absence of cystic fibrosis. *Lancet* 1994;**344**:1473-1474. | Including only CBAVD cases |
| 3. | Gallati S, Hess S, Galie-Wunder D, Berger-Menz E, Bohlen D. Cystic fibrosis transmembrane conductance regulator mutations in azoospermic and oligospermic men and their partners. *Reprod Biomed Online* 2009;**19**:685-694. | Full text/detailed data unavailable |
| 4. | Jezequel P, Chauvel B, Le Treut A, Le Gall JY, David V, Le Lannou D, Blayau M. Identification of a novel mutation in CFTR gene exon 8 (L375F) in a CUAVD phenotype. *Hum Genet* 1996;**97**:548-549. | Family study/case report |
| 5. | Le Lannou D, Jezequel P, Blayau M, Dorval I, Lemoine P, Dabadie A, Roussey M, Le Marec B, Legall JY. Obstructive azoospermia with agenesis of vas deferens or with bronchiectasia (Young's syndrome): a genetic approach. *Hum Reprod* 1995;**10**:338-341. | Full text/detailed data unavailable |
| 6. | Lissens W, Mercier B, Tournaye H, Bonduelle M, Ferec C, Seneca S, Devroey P, Silber S, Van Steirteghem A, Liebaers I. Cystic fibrosis and infertility caused by congenital bilateral absence of the vas deferens and related clinical entities. *Hum Reprod* 1996;**11 Suppl 4**:55-78; discussion 79-80. | Review/conference abstract |
| 7. | Meschede D, Dworniczak B, Nieschlag E, Horst J. Genetic diseases of the seminal ducts. *Biomed Pharmacother* 1998;**52**:197-203. | Review/conference abstract |
| 8. | Ocak Z, Uyetuork U, Dincer MM. Clinical and prognostic importance of chromosomal abnormalities, Y chromosome microdeletions, and CFTR gene mutations in individuals with azoospermia or severe oligospermia. *Turk J Med Sci* 2014;**44**:347-351. | Full text/detailed data unavailable |
| 9. | Patrizio P, Zielenski J. Congenital absence of the vas deferens: a mild form of cystic fibrosis. *Mol Med Today* 1996;**2**:24-31. | Review/conference abstract |
| 10. | Patrizio P, Asch RH, Handelin B, Silber SJ. Aetiology of congenital absence of vas deferens: genetic study of three generations. *Hum Reprod* 1993;**8**:215-220. | Including only CBAVD cases |
| 11 | Patrizio P, Ord T, Silber SJ, Asch RH. Cystic fibrosis mutations impair the fertilization rate of epididymal sperm from men with congenital absence of the vas deferens. *Hum Reprod* 1993;**8**:1259-1263. | Full text/detailed data unavailable |
| 12. | Patrizio P, Silber S, Ord T, Balmaceda JP, Asch RH. Two births after microsurgical sperm aspiration in congenital absence of vas deferens. *Lancet* 1988;**2**:1364. | Review/conference abstract |
| 13 | Pichler R, Oswald J, Glodny B, Skradski V, Aigner F, Rehder P. Unilateral renal agenesis with absent ductus deferens, epididymis and seminal vesicle: incidental finding in a 22-year-old patient with maldevelopment of the mesonephric duct. *Urol Int* 2011;**86**:365-369. | Family study/case report |
| 14 | Radpour R, Gourabi H, Gilani MA, Dizaj AV. Correlation between CFTR gene mutations in Iranian men with congenital absence of the vas deferens and anatomical genital phenotype. *J Androl* 2008;**29**:35-40. | Duplication/overlapping data |
| 15 | Radpour R, Gilani MA, Gourabi H, Dizaj AV, Mollamohamadi S. Molecular analysis of the IVS8-T splice variant 5T and M470V exon 10 missense polymorphism in Iranian males with congenital bilateral absence of the vas deferens. *Mol Hum Reprod* 2006;**12**:469-473. | Duplication/overlapping data |
| 16 | Sharma H, Mavuduru RS, Singh SK, Prasad R. Heterogeneous spectrum of mutations in CFTR gene from Indian patients with congenital absence of the vas deferens and their association with cystic fibrosis genetic modifiers. *Mol Hum Reprod* 2014;**20**:827-835. | Full text/detailed data unavailable |
| 17 | Silber S, Ord T, Borrero C, Balmaceda J, Asch R. New treatment for infertility due to congenital absence of vas deferens. *Lancet* 1987;**2**:850-851. | Review/conference abstract |
| 18 | Weiske WH, Salzler N, Schroeder-Printzen I, Weidner W. Clinical findings in congenital absence of the vasa deferentia. *Andrologia* 2000;**32**:13-18. | Full text/detailed data unavailable |
| 19 | Yang XJ, Yuan P, Wu X, Zhang H, He QQ, Zhang Y. [Detection of CFTR gene mutations in azoospermia patients with congenital unilateral absence of the vas deferens]. *Zhonghua Nan Ke Xue* 2015;**21**:229-233. | Duplication/overlapping data |
| 20 | Zeng G, Mei H, Zhuang G, Li M. [Study of CFTR gene mutation in Chinese CUAVD patients]. *Zhonghua Yi Xue Yi Chuan Xue Za Zhi* 2000;**17**:241-243. | Duplication/overlapping data |

**Supplementary Table S3** Data extraction of the included studies (N = 23).

| **Authors (Year)** | **Case frequency of *CFTR* variants** | | |  | **Frequency of common genotype/allele** | | | | |  | **Frequency of 5T allele** | |
| --- | --- | --- | --- | --- | --- | --- | --- | --- | --- | --- | --- | --- |
|  | **At least one** | **Two** | **One** |  | **F508del/5T** | **F508del/R117H** | **F508del** | **5T** | **R117H** |  | **Non-CAVD** | **Normal** |
| (Yang, Sun *et al.*, 2015) | 3 | 1 | 2 |  | 0/5 | 0/5 | 0/10 | 3/10 | 0/10 |  | NA | NA |
| (Chiang, Lin *et al.*, 2013) | 1 | 0 | 1 |  | NA | NA | NA | 1/2 | NA |  | NA | NA |
| (Schwarzer and Schwarz, 2012) | 0 | 0 | 0 |  | 0/8 | 0/8 | 0/16 | 0/16 | 0/16 |  | NA | NA |
| (Sharma, Acharya *et al.*, 2009) | 5 | 1 | 4 |  | 0/7 | 0/7 | 2/14 | 4/14 | 1/14 |  | 5/100 | NA |
| (Radpour, Gourabi *et al*., 2007) | 5 | 0 | 5 |  | 0/7 | 0/7 | 0/14 | 3/14 | 1/14 |  | NA | NA |
| (Danziger, Black *et al.*, 2004) | 0 | 0 | 0 |  | 0/1 | 0/1 | 0/2 | 0/2 | 0/2 |  | 0/168 | NA |
| (Grangeia, Niel *et al.*, 2004) | 3 | 0 | 3 |  | 0/4 | 0/4 | 1/8 | 2/8 | 0/8 |  | NA | NA |
| (Kolettis and Sandlow, 2002) | 2 | 0 | 2 |  | 1/6 | 0/6 | 2/12 | 1/12 | 0/12 |  | 8/228 | 7/44 |
| (Robert, Bey-Omar *et al.*, 2002) | 3 | 0 | 3 |  | 0/6 | 0/6 | 0/12 | 3/12 | 0/12 |  | NA | NA |
| (Larriba, Bassas *et al.*, 2001) | 3 | 2 | 1 |  | 0/4 | 0/4 | 0/8 | 1/8 | 0/8 |  | NA | NA |
| (Attardo, Vicari *et al.*, 2001) | 1 | 1 | 0 |  | 0/1 | 0/1 | 0/2 | 1/2 | 0/2 |  | NA | 2/60 |
| (Zeng, Mei *et al.*, 2000) | 1 | 0 | 1 |  | 0/1 | 0/1 | 0/2 | 0/2 | 0/2 |  | NA | NA |
| (Jézéquel, Dubourg *et al.*, 2000) | 3 | 3 | 0 |  | 0/3 | 1/3 | 1/6 | 0/6 | 1/6 |  | NA | NA |
| (Casals, Bassas *et al.*, 2000) | 6 | 5 | 1 |  | 0/14 | 0/14 | 2/28 | 5/28 | 0/28 |  | NA | NA |
| (Castellani, Bonizzato *et al.*, 1999) | 2 | 1 | 1 |  | 0/3 | 0/3 | 3/6 | 0/6 | 0/6 |  | 20/400 | NA |
| (Boucher, Creveaux *et al.*, 1999) | 2 | 1 | 1 |  | 1/2 | 0/2 | 1/4 | 2/4 | 0/4 |  | NA | NA |
| (Dörk, Dworniczak *et al.*, 1997) | 3 | 2 | 1 |  | 1/5 | 1/5 | 3/10 | 1/10 | 1/10 |  | NA | 3/78 |
| (Schlegel, Shin *et al.*, 1996) | 3 | 0 | 3 |  | NA | 0/12 | 2/24 | NA | 0/24 |  | NA | NA |
| (Mickle, Milunsky *et al.*, 1995) | 8 | 0 | 8 |  | NA | 0/16 | 4/32 | NA | 2/32 |  | NA | NA |
| (Jarvi, Zielenski *et al.*, 1995) | 1 | 0 | 1 |  | 0/2 | 0/2 | 1/4 | 0/4 | 0/4 |  | NA | NA |
| (Casals, Bassas *et al.*, 1995) | 1 | 0 | 1 |  | 0/6 | 0/6 | 1/12 | 0/12 | 0/12 |  | NA | 1/36 |
| (Chillón, Casals *et al.*, 1995) | 3 | 0 | 3 |  | NA | NA | NA | 3/24 | NA |  | NA | NA |
| (Culard, Desgeorges *et al.*, 1994) | 1 | 0 | 1 |  | NA | 0/1 | 0/2 | NA | 0/2 |  | 5/92 | 1/10 |

Notes: NA: not applicable; Normal: normal controls; Non-CAVD: azoospermia patients of none congenital absence of the vas deferens.

**Supplementary Table S4** Summary for meta-analyses and test of publication bias in CUAVD.

| **Outcomes** | **No. of studies** | **Events/Total** | **Pooled effect size, 95%CI** | **Test of Heterogeneity** | | **Tests of Publication Bias** | |
| --- | --- | --- | --- | --- | --- | --- | --- |
|  |  |  |  | ***I*^2^ (%)** | ***P*** | **Begg's *P*** | **Egger's *P*** |
| At least one variant | 23 | 60/141 | 0.46, [0.31, 0.62] | 53% | <0.01 | 0.03 | 0.01 |
| Two variants | 23 | 17/141 | 0.05, [0.00, 0.17] | 54% | <0.01 | <0.01 | 0.02 |
| One variant | 23 | 43/141 | 0.27, [0.15, 0.41] | 39% | 0.03 | 0.42 | 0.15 |
| F508del/5T genotype | 18 | 3/99 | 0.00, [0.00, 0.02] | 0% | 0.96 | <0.01 | 0.01 |
| F508del allele | 21 | 23/256 | 0.04, [0.01, 0.08] | 30% | 0.10 | 0.09 | 0.12 |
| 5T allele | 20 | 30/224 | 0.09, [0.03, 0.17] | 44% | 0.02 | 0.28 | 0.14 |
| F508/R117H genotype | 21 | 2/128 | 0.00, [0.00, 0.00] | 0% | 0.99 | <0.01 | <0.01 |
| R117H allele | 21 | 6/256 | 0.00, [0.00, 0.01] | 0% | 0.95 | 0.01 | 0.08 |
| RA in CUAVD | 15 | 35/129 | 0.22, [0.13, 0.32] | 0% | 0.66 | 0.08 | 0.03 |
| RA in CBAVD | 14 | 41/724 | 0.05, [0.02, 0.08] | 52% | 0.01 | 0.32 | 0.17 |
| Pooling OR of RA risk | 13 | 26/97; 41/612 | 4.85, [2.87, 8.20] | 0% | 0.58 | 0.58 | 0.06 |

Notes: NA: not applicable; NC: normal controls; non-CAVD: azoospermia patients of none congenital absence of the vas deferens.

**Supplementary Table S5** *CFTR* variant among CUAVD patients complicated with RA.

| **Study** | **RA (+)** | |  | **RA (-)** | |
| --- | --- | --- | --- | --- | --- |
|  | ***CFTR* (+)** | ***CFTR* (-)** |  | ***CFTR* (+)** | ***CFTR* (-)** |
| Yang *et al.* (2015) | 0 | 1 |  | 3 | 2 |
| Chiang *et al.* (2013) | 0 | 1 |  | 1 | 0 |
| Schwarzer and Schwarz (2012) | 0 | 5 |  | 0 | 8 |
| Sharma *et al.* (2009) | 0 | 3 |  | 5 | 2 |
| Danziger *et al.* (2004) | 0 | 0 |  | 0 | 1 |
| Grangeia *et al.* (2004) | 0 | 0 |  | 3 | 1 |
| Kolettis and Sandlow (2002) * | 0 | 4 |  | 2 | 4 |
| Robert *et al.* (2002) | 0 | 1 |  | 3 | 3 |
| Attardo *et al.* (2001) | 0 | 0 |  | 1 | 0 |
| Casals *et al.* (2000) | 3 | 7 |  | 3 | 11 |
| Castellani *et al.* (1999) | 0 | 0 |  | 2 | 1 |
| Boucher *et al.* (1999) | 0 | 0 |  | 2 | 0 |
| Dörk *et al.* (1997) | 0 | 0 |  | 3 | 2 |
| Schlegel *et al.* (1996) * | 0 | 5 |  | 3 | 4 |
| Mickle *et al.* (1995) | 0 | 5 |  | 8 | 8 |
| Total | 3 | 32 |  | 39 | 47 |

Notes: RA, renal abnormality; +, positive; -, negative; *, eight CUAVD patients complicated with RA declined to perform *CFTR* variant screening.
